# Maternal care practices among the ultra poor households in rural Bangladesh: a qualitative exploratory study

**DOI:** 10.1186/1471-2393-11-15

**Published:** 2011-03-01

**Authors:** Nuzhat Choudhury, Syed M Ahmed

**Affiliations:** 1 Research and Evaluation Division, BRAC Centre, 75 Mohakhali, Dhaka 1212, Bangladesh; 2 Nutrition Programme, Clinical Science Division, International Centre for Diarrhoeal Disease Research Bangladesh (ICDDR,B), 68 Shaheed Tajuddin Ahmed Sharani, Mohakhali, Dhaka 1212, Bangladesh

## Abstract

**Background:**

Although many studies have been carried out to learn about maternal care practices in rural areas and urban-slums of Bangladesh, none have focused on ultra poor women. Understanding the context in which women would be willing to accept new practices is essential for developing realistic and relevant behaviour change messages. This study sought to fill in this knowledge gap by exploring maternal care practices among women who participated in a grant-based livelihood programme for the ultra poor. This is expected to assist the designing of the health education messages programme in an effort to improve maternal morbidity and survival towards achieving the UN millennium Development Goal 5.

**Methods:**

Qualitative method was used to collect data on maternal care practices during pregnancy, delivery, and post-partum period from women in ultra poor households. The sample included both currently pregnant women who have had a previous childbirth, and lactating women, participating in a grant-based livelihood development programme. Rangpur and Kurigram districts in northern Bangladesh were selected for data collection.

**Results:**

Women usually considered pregnancy as a normal event unless complications arose, and most of them refrained from seeking antenatal care (ANC) except for confirmation of pregnancy, and no prior preparation for childbirth was taken. Financial constraints, coupled with traditional beliefs and rituals, delayed care-seeking in cases where complications arose. Delivery usually took place on the floor in the squatting posture and the attendants did not always follow antiseptic measures such as washing hands before conducting delivery. Following the birth of the baby, attention was mainly focused on the expulsion of the placenta and various maneuvres were adapted to hasten the process, which were sometimes harmful. There were multiple food-related taboos and restrictions, which decreased the consumption of protein during pregnancy and post-partum period. Women usually failed to go to the healthcare providers for illnesses in the post-partum period.

**Conclusion:**

This study shows that cultural beliefs and norms have a strong influence on maternal care practices among the ultra poor households, and override the beneficial economic effects from livelihood support intervention. Some of these practices, often compromised by various taboos and beliefs, may become harmful at times. Health behavior education in this livelihood support program can be carefully tailored to local cultural beliefs to achieve better maternal outcomes.

## Background

Nearly one quarter to one third of Bangladesh's population lives under extreme poverty [1]. To improve the maternal health indicators it is necessary to develop interventions that meet the health needs of extremely poor women. These extremely poor households, henceforth ultra poor households, have few or no asset base, are highly vulnerable to any shock (e.g., natural disaster, disability of an income-earner or illnesses involving costly care), and mainly depend on seasonal wage-labour for survival. They are characterized by their inability to participate fully in social and economic activities which make them vulnerable to differential treatment of the society [2]. As a result, they are often excluded from government and even from the microcredit-based non-governmental poverty reduction programmes [3].

To reach the ultra poor in rural Bangladesh who could not benefit from conventional microcredit programmes, BRAC (an indigenous non-government development organization) implemented an integrated, grant-based programme (comprising asset transfer, skills training, enterprise support, weekly stipend and healthcare support to mitigate income-erosion effect of illnesses) during 2002-2006 called *Challenging the Frontiers of Poverty Reduction *Phase I (CFPR-I) [3]. Five inclusion criteria (dependent upon female domestic work or begging as income source; possessing ≤10 decimals of land; no male adult active member in the household; children of school going age engaged in paid work; and absence of productive assets in the household) and three exclusion criteria (no adult woman in the household who is able to work; participating in microfinance; and beneficiary of government/NGO development project) were used to identify the ultra poor households for the BRAC CFPR programme. Only those surveyed households who met at least three of the five inclusion criteria and none of the exclusion criteria, were finally selected as the beneficiaries of the CFPR programme. Thus the study population refers to the lowest wealth quintile in a community. A series of evaluations also showed positive impact from the programme including improved income, food security and nutritional status, and health knowledge and behaviour [4-6]. Drawing on the learning and experiences from the first phase (CFPR-I), the five-year second phase of this programme (CFPR-II) started in 2007 with an even greater scale and scope [7].

Bangladesh is one of the low- income countries where poorer groups use less health care and there exists a poor-rich inequality in maternity care and maternal mortality [2,8]. Apart from this poor-rich inequality, social and cultural beliefs and practices regarding motherhood and childrearing also have significant influence on maternal health [9-13]. As a result, these poorest-of-the-poor women suffer a greater burden of ill health including maternal health [3]. They are marginalized compared to national rural average in terms of accessing antenatal care (ANC) (37% for ultra poor women and 60% national rural women), institutional delivery (5% from lowest wealth quintile and 15% national rural average), receiving iron- supplementation during last pregnancy (53% for ultra poor and 55% national rural women), and use of contraceptive prevalence (63% for ultra poor and 80% ever-married women in Bangladesh) [7,14]. These maternal health and health care indicators are also important determinants of neonatal survival [15], which is an important contributing factor for infant survival. Different studies have been carried out to learn about maternal care practices in the rural areas [9-11,13,16,17] or urban-slums of Bangladesh [12,18], but none of them specifically focused on the marginal population such as the ultra poor households. Understanding the context in which women and their families would be willing to accept new practices, i.e., knowing what changes they would make under what conditions is essential for crafting realistic and relevant behaviour change messages. This study sought to fill in this knowledge gap by qualitative exploration of the existing maternal care practices during pregnancy, delivery and post-partum period among women of the ultra poor households who were included in the CFPR II programme (2007-2012). It also attempted to highlight the underlying reasons for doing such maternal care practices. This, in turn, is expected to inform the programme interventions which would hopefully improve maternal morbidity and survival, and achieve the UN Millennium Development Goal 5 (MDG 5).

## Methods

A qualitative exploratory framework was used to collect maternal care practice from women in ultra poor households. The sample included both currently pregnant women who have had at least one previous childbirth, and lactating mothers who are living in the CFPR-II programme areas [7]. Rangpur and Kurigram districts in the northern part of Bangladesh were selected for data collection. Later, eight administrative offices of CFPR-II programme from the above two districts were selected where CFPR-II baseline survey was conducted [7]. To identify the respondents, two anthropologists with the help of the researchers have visited each office and listed the names of the CFPR-beneficiary households, the address, and the availability of eligible respondents using a household listing form. From each of these lists, 2-3 households have been randomly selected for conducting the interviews. Thus, 20 women- 12 lactating mothers and 8 currently pregnant women were selected for the study.

The study has passed through the institutional review process at BRAC Research and Evaluation Division (by Internal Review and Publication Committee) for ethical approval. The ethical clearance was also obtained from Bangladesh Medical Research Council (BMRC) for the entire five-year research project to evaluate the CFPR-II programme. Data were collected after obtaining informed verbal consent from the respondents who were hesitant about signing any document. The written consent form was read and explained to the respondent. When the investigator was satisfied that the respondent understood it including its implications, and agreed to participate, only then she was selected for the in-depth interview. Anonymity of the respondents was maintained at all stages of data analysis.

Data were collected through in-depth interview in Bangla following a flexible interview guide during October-December 2007. The interviews were conducted by two experienced anthropologists under supervision of the researchers. The in-depth interview guide comprised three sections; the first section included socioeconomic information and cultural background of extreme poor households; the second section included maternal care practices and rituals which covered pregnancy care, delivery care, and postpartum care; and the last part included their beliefs regarding the care practices and recommendations. The interviews lasted between 60 and 90 minutes. Throughout the process, many loosely structured informal discussions took place, because, in a communal setting, it was difficult to conduct one-to-one interviews and discuss their individual perceptions of practices.

All the interviews were recorded and transcribed on the same day. The transcripts were analyzed thematically. Transcripts were reviewed to develop a code list for the topics related to the research questions. Codes were applied manually by the interviewers. Text pertaining to the codes was organized in a matrix and translated into English. No software was used for the analysis.

## Results

### Respondent's profile

The majority (15 out of 20 respondents) of the women were in their twenties, two were below 20 years of age while three were in their thirties. Most of the interviewees were Muslim. Non-farming labour, like rickshaw pulling, was one of the most common occupations of the household heads. Most of them lived in poor hygienic condition as revealed by the use of proper sanitation (with or without water seal latrine or pit latrine), water access, and overall household condition. At the time of data collection, all the respondents were in their initial phase of the CFPR-II programme.

### Pregnancy care

Almost all the women reported about becoming aware of their pregnancy when they experienced amenorrhoea, nausea and vomiting, loss of appetite and weakness. Most of them could identify their pregnancy within the first 2-3 months. According to them, pregnancy identification and its subsequent care was seen as a normal event which did not require any additional medical intervention unless significant complications arose during this period. Two women were found to have had no menstruation history after delivery of the last baby till conception of the next one.

#### Antenatal care

Confirmation of pregnancy was considered by the women as the most important part of antenatal care and eight out of 20 women went to the nearby health facilities for pregnancy tests. Reportedly, the most common service that a pregnant woman received in ante-natal clinics was iron supplementation. However, most of them did not take all the tablets dispensed because they perceived the tablets to be tasteless (or have bad taste) and made the stool black. None of them took iron tablets for more than two months during their last pregnancy. Most of these women opined that antenatal care provided no benefit to them or their child. Monetary constraints, absence of knowledge about the need of services, and restrictions on the movement of women were also cited as reasons for not accessing antenatal care.

#### Social exclusion

The following finding is not unique but typical of the broad experience of ultra poor women living in the rural areas of Bangladesh:

*"I was avoiding health worker because she would scold me if she would have heard about my 4*^*th *^*pregnancy. She used to give me birth control pills. So, I did not meet her and inform her*." (A lactating mother of 27 years old)

In two cases, the women reported that the respective health worker came to their house to give birth control pills. However, neither of them came out of their respective houses. The use of harsh words and low tolerance level of the health workers discouraged the women to use the services provided by these health facilities for antenatal care. These women perceived that they were treated in such a manner by the health worker because of their low socioeconomic status.

*"I heard that health service from government facility is free of charge but when I went to the health facility I was asked to make a card for Tk. 20.Which services are then considered to be free of charge?" *(A pregnant woman of 21 years old)

#### Nutrition during pregnancy

Women reported that the reasons for decreasing consumption of food during pregnancy were mostly related to aversion to specific food, followed by lack of monetary power to purchase specific food that extremely poor households usually consumed (like rice, potato, and small fish). However, few women also reported increasing consumption of food during pregnancy. The reasons given for this varied from those given for decreasing consumption. The most frequently cited reason was 'feel like eating more.' 'Craving for a specific food 'was also cited as a reason for increased consumption of some food such as molasses-made drink, rice with green chilies, and milk. Two of the women mentioned that the increased food intake was directly related to improved health of the mother or the baby. This tended to be where husbands and other family members were helpful and better informed.

*"I know that eating more food is necessary when there is a baby in womb. But I am poor, how can I afford it?" *(A pregnant woman of 21 years old)

Some food, like ducks, pigeons, beef and Hilsha fish were considered as 'hot' and were restricted during pregnancy. Some fish like *Taki*, *Chanda *and *Puti*, which were within their affordability, were also restricted during pregnancy. There were no restrictions reported in consuming fruits among the extreme poor households.

#### Restrictions and mobility during pregnancy

It is generally believed among the extreme poor households that evil spirits are more active in the evening, at noon and at night, so pregnant women avoided leaving their houses during those times. Walking through graveyards was also thought to be harmful for pregnant women. If they did so, they tied up their hair and covered their heads with veils.

*"Evil spirits could cause miscarriage of the fetus,that is why I did not go out in prayer time*" (A lactating mother of 31 years old)

A few women reported their beliefs about carrying a piece of iron which would ensure protection. Matches were also reported to be effective in keeping away the evil gaze of the spirits. Most of the respondents mentioned that lunar and solar eclipses could affect pregnant women. They reported (those who got eclipse during last pregnancy) that they had stayed inside the household, walked near the home or inside the home, but had never laid down on the bed during eclipses. They also reported certain restrictions during this period - like they did not eat or cook, cut, and twist anything, as they perceived that the child would be born with a cleft palate or with deformed features. Many of the women reported that elderly family members and husbands were the main informants as to when there would be a lunar or solar eclipse. In any case, restrictions in movement had never been imposed by any health providers but rather from the elderly women of the family.

#### Support from husband

Present study found that in the midst of poverty, the husband could play a positive role in taking care of his wife during the pregnancy period. An illustrative case:

*All that Mamtaz's husband can claim as his own are the homestead land and the house. He inherited three decimals of land from his father on which he has built a house to live in. He is physically disabled. He does not have any source of income other than the 2/3 kg of rice that he gets from begging door to door. From that amount, Mamtaz keeps whatever is required for the household and sells the remaining to buy other necessities such as salt, vegetables to run her family. Sometimes when her husband is unable to go for begging, Mamtaz would go to other people's houses for work during pregnancy. Her husband did not wish for her to work at other people's houses during her pregnancy and expressed that whatever he earned through begging was enough for them to sustain. But still, when he was not at home and someone called her for assistance, she would go to their houses to boil paddy or for maintenance of floors in exchange of one or half a kg of rice*. (A pregnant woman of 23 years old)

Women considered this attitude of their husbands as a positive attitude if the women were too weak to work or continue the usual household work. In three cases, women consulted with their husbands and jointly decided to stop their other child's schooling so that the child would rear the animals and the women could rest during their pregnancy. Overall, during pregnancy, women reported that husbands and other family members helped them in doing heavy work. Activities such as fetching water, boiling and husking rice, lifting heavy cooking utensils and preparing food for animals were generally regarded as heavy work.

#### Birth preparedness

Birth preparedness for this present study included selecting a skilled birth attendant, arranging delivery kit needed for a safe birth, identifying where to go in case of emergency, and arranging necessary money and transport for delivery. We found only one woman who had a birth plan (took into consideration all the outlined stages of the preparation) before delivery. Most women did not contact the birth attendant who is locally known as traditional birth attendants (TBAs or *dais*) in advance because they thought TBAs could make some *jadutona *(Black magic) in advance during pregnancy so that without their presence delivery would not occur and that there were greater chances to pay more for delivery.

*"After I started having pain on the morning of the 3rd day, my husband went out to call the TBAs*. *TBAs was not informed beforehand as she took more money if asked to stay for a longer period of time. This TBA perhaps is trained. Most of the children in this locality were delivered by her *(A lactating mother of 19 years old)

Few families, especially the husbands, could put aside some money for delivery purpose. One husband had financial constraints, so he saved 40 kg of rice. Twelve women said that they only collected some old clothes, which they had kept separately, but they had not stitched any new dresses or *Kathas *(local quilt covering) for the arrival of the new baby. The women believed that it was bad to buy new clothes or make too many plans in advance for the new arrival as it could bring bad luck. Moreover, they were not sure whether the coming child would survive or not. Money spent on her/him was considered to be unnecessary. Women assumed that transportation would be available either from a family member or from a neighbour when needed and, as such, did not plan for the transportation in advance.

### Delivery care

We found that after the initiation of labour pain, elderly women usually took matters into their own hands by taking various steps and observing certain rituals to facilitate early delivery of the baby. Women reported that sometimes relatives fed them *pora pani *(enchanted water) to boost up their mental strength, which referred to the psychological aspect of the expectant mothers as being strong enough to face the labour pain. For providing energy to the delivering mother as well as to intensify the labour pain, five mothers said that they had received saline with an injection (oxytocin) from a neighbouring *Pallichikitshok *(Village doctor).

#### Place of birth and attendance at delivery

Most of the deliveries took place at home (19 out of 20). The TBA was usually called after the onset of strong labour pain. TBA or friends/relatives were the most common persons to be present as the birth attendant during delivery. Almost all the deliveries took place on the floor. Four women even gave births on just the bare floor, but for the rest, the deliveries took place on spread out cloths or jute sacks or straw. Delivery was not carried out on beds in order to avoid spoiling it. According to the women, few materials like straw, polythene, etc. if placed on the floor, made cleaning and disposing of impure blood and placenta much easier. Ten out of the 20 women reported that their preferred position was squatting while giving birth. However, this may have, and often did change, as labour progressed. For three of the women, squatting position was found to be more painful than lying. Usually, the position to be adopted was often decided upon discussion with the TBA and other female relatives. No cases were found where any male members of the household were involved during delivery.

Health system factors such as staff attitudes from healthcare, either public or private, also had an impact on maternal care practice. Poorly attitude of the healthcare staffs were perceived to exist in most health facilities; these included usage of abusive language, denial of providing services, lacking compassion in general and refusal to assist properly.

*"I went to government facility for antenatal care. The concerned person told me I might need cesarean, and I would die if I did not go to the hospital. Are these words good to tell someone who is pregnant?" *(A pregnant woman of 17 years old)

The mothers were asked whether or not the birth attendant had washed their hands with soap before delivery, and whether or not the instrument used for cutting the umbilical cord and the thread used for tying the cord were boiled before use. Washing hands before conducting the delivery was very low. Boiling the blade was high, but it was reported that they did not boil the thread. Women reported that at times, the TBAs kept the thread aflame only and did not boil it if the thread was new.

#### Practices to speed up the delivery of placenta

It was found that the focal point of attention after birth of the baby was mainly on the removal/expulsion of the placenta, as the placenta was believed to have spiritual value. It was believed that after the baby's birth, if the placenta was not delivered quickly, the mother would be in danger. Nine women believed that the placenta could move up to the throat and choke them to death if not removed promptly. To release the placenta after delivery or in cases where there was a delay in the process, TBAs or relatives were found to massage the abdomen of the women, gag her with her hair or give kerosene oil or onion juice to induce vomiting which was believed to help expel the placenta through abdominal contractions. A woman reported that the TBA wiped her chest with a dirty cloth (which was used in mud cleaning) and this was followed by expulsion of the placenta. Treatment of placenta was sometimes considered a priority than treatment of the newborn immediately after birth. It was believed that placenta should be buried in the dry soil so that the child would not suffer from any cold or cough at a later stage. To save some money, some women preferred to cut their umbilical cord themselves. Eight out of 20 women cut their umbilical cord in their last delivery. If any other woman had cut the umbilical cord for them, then they had to pay a minimum of Tk. 20 (US$ 0.30).

### Post-partum care

During the post-partum period, especially during the first 5-9 days of isolation, the mothers reported various dietary restrictions imposed on them which deprived them of proper nutrition intake. Most food available in these extreme poor households was thought to be inappropriate during lactation. In the case of four women, no food at all was allowed for consumption during the first few days after delivery, and commonly no food was given at all during the first day after delivery to allow healing of the birth passage. Moreover, women were considered to be impure during this time. They were not allowed to touch any food for preparing meals for other family members. In-depth interviews revealed that the mothers-in-law and the elders played a dominant role in deciding what foodstuffs the mother could eat. Shujata's child, who was not even a month old, lived with her family consisting of her husband, mother and an elder sister. She said:

*"We all live together, use the same kitchen but have separate rooms. Since the child's delivery, my mother and sister prepare the food as the newly delivered women (Poaati ma) are not supposed to cook till 40 days after the delivery because their body is considered to be impure. People wouldn't like it if I cook and it's not good for me even. My mother brings me food in my room and gives me lesser than my usual intake of food so that I don't fall ill. Poaati ma (lactating mother) should eat as less as possible till her umbilical cord dries up. It does not matter if I'm still hungry and feel weak and as long as I don't have to spend money for doctor's visit. It does not harm if you follow the elder women. I have the whole life to myself to eat more. So it's fine if I eat a little less the 1st 1-2 months. I prefer weakness to illness*. (A lactating mother of 23 years old)

These women did not seem to have any problem with this imposed dietary restriction because their economic condition did not allow them to buy animal source food like beef, chicken anyway.

The most common items eaten during the post-partum period included rice, smashed potato with spices, raw tea, green banana, black cumin, poppy seed, fenugreek leaves etc. These are believed to keep the stomach cool and initiate the production of breast milk. Whatever special food was consumed during post-partum period, it was reported to be only for a few days while the imposed food restrictions continued for a longer time i.e., 21-40 days. Opinions given on the intake of spicy food were mixed. It was given for the first few days for healing of the birth passage but later on, the same was restricted to avoid heart-burning.

Women reported that during their last pregnancy they felt weak with severe body-aches (11 out of 20 interviews) after delivery. It lasted for one to three weeks. None of them had gone to any health providers for seeking any service for this weakness and body-ache. These women reasoned that they did not go for the check-up because they were not even aware about the availability of the post-partum check-up. Four women reported that their husbands or mothers had gone to pharmacies to fetch vitamin tablets or saline when they had expressed about their weaknesses. This weakness was however considered to be a common part of their post-partum life. To quote one woman:

*"It is normal to have some bodyache and fever after delivery, these would be cured automatically*" (A lactating mother of 24 years old)

## Discussion

There is a dearth of information on maternal care practices of the marginalized women from ultra poor households in rural Bangladesh. This study attempted to fill in some of this knowledge gap through qualitative in-depth interviews of a group of women participating in a grant-based livelihood development programme for the ultra poor. Findings regarding practices were very similar to what is known for the poor rural women [9-11,13,16,17] or women from the urban slums [12,18] which clearly indicate the dominance of the powerful influence of culture and tradition in this area.

It is well documented that demographic characteristics, affluence and socio-cultural factors play a major role in maternal care practices [2]. The present study has also emphasized and reconfirmed the fact that dire poverty and social exclusion create an environment which pushes mothers away from antenatal or post-natal care services. Antenatal care is one of the "four pillars" of safe motherhood, as formulated by the Maternal Health and Safe Motherhood Programme, Division of Family Health of the World Health Organization (WHO) [19]. Literature suggests that home visits by community-based health workers can help to reduce neonatal mortality by ensuring identification of pregnant women, and by ensuring optimal maternal health through both antenatal and postnatal care visits to their homes [13,15,20,21]. When multiple health services are provided by one single person in a community, problems can arise. For example, it was found from the present study that since the same individual was responsible for providing contraceptive pills as well as for antenatal care, the women felt shy and sometimes scared to share their pregnancy news with the health care provider in fear of being scolded for discontinuation of the contraception. This tendency of healthcare providers, as reported elsewhere, [2] to reprimand mothers in some situations may act as a discouraging factor for the ultra poor women for using the healthcare services, and this may cause less number of pregnant women to be identified on the whole. This situation will only worsen if a woman experiences constraints on seeking care for herself by the involvement of other family members in the decision-making process. This involvement of other family members is very common in Bangladesh as found in other studies [2,12,13,16]. There is a need to sensitize health workers to the needs of the pregnant mothers so that the beneficiaries are not scared of them. Women should be able to share their problems easily with the healthcare providers. However, it is encouraging to note that no traditional beliefs were reinforced by the healthcare providers in the community.

In South Asia and even in some of the African countries, only a small proportion of women perform deliveries in healthcare facilities [14-16,21,22]. Birth preparedness in this region is also low [12,20]. Financial constraints coupled with traditional beliefs and rituals have been seen to delay and sometimes stop them altogether from taking any prior preparation for childbirth. This clearly means that there is a dire need to ensure a high level of awareness among pregnant women to address the importance of planned delivery. Beliefs and rituals also have an effect on maternal nutrition. The ultra poor households' per capita mean food consumption is low compared to national average intake [3,4,14]. Half of the ultra poor women are suffering from malnutrition [4], which often becomes worse because of food taboos during pregnancy and post-partum period. However, commonly taken drinks and food such as raw tea, black cumin, poppy seed, fenugreek leaves, *neem *leaves have health benefits beyond basic nutrition [23]. As the BRAC CFPR II programme is reaching households to deliver messages on health issues, other family members or neighbours can always be advised not to impose any dietary restrictions during pregnancy and most importantly in the post-partum period.

It was reported that the deliveries were conducted mostly by keeping the mothers in squatting position which is similar with the findings of other studies in Bangladesh [10,20]. The present study reveals the existence of various beliefs and practices regarding the expulsion of placenta which was often risky as found in other studies [11,12,16]. Adopting these malpractices along with the post-partum confinement of mothers in ultra poor households as found in this culture, could be major factors responsible for the delay or prevention of care-seeking outside the home. According to the respondents in this study, care giving is considered to be offered by a female within the households. Men become involved in maternal care provision in those situations where there is no able-bodied female to take over the role. A husband's involvement in particular situations like 'not allowing any heavy work during pregnancy' and 'going to the pharmacy to bring vitamin and supplementation', could shift some of the care burden and distribute it more equally within the family. In both the cases, a positive outcome was found. For instance, the pregnant woman was able to take rest during pregnancy and could take vitamin supplementation in post-partum period. Focus can be imposed on increasing the role of men/boys in care provision beyond the traditionally-defined role.

These findings are based on self-reported maternal care practices, and may therefore differ from actual practices. Although we intended to use the participants' observation to obtain and validate data, this was not possible due to resource constraints. However, given the consistency of findings in qualitative interviews and other quantitative studies [16,17,24], we are confident that the findings represent actual practices.

## Conclusions

This paper attempts to outline the potential areas for programme interventions to improve maternal morbidity and mortality in rural areas of Bangladesh towards achieving the Millennium Development Goal 5. This study shows that cultural beliefs and norms have a strong influence on maternal care practices among the ultra poor households, and override the beneficial economic effects from livelihood support intervention. Some of these practices, often compromised by various taboos and beliefs, may become harmful at times. Health behavior education in this livelihood support program can be carefully tailored to local cultural beliefs to achieve better maternal outcomes. Furthermore, a quantitative research could be carried out to know the accurate level and extent of disadvantages suffered by the ultra poor women compared to other groups.

## Competing interests

The authors declare that they have no competing interests.

## Authors' contributions

NC and SMA conceived this qualitative study and participated in its design. NC was involved in coordination, data collection, and data analysis. Both authors interpreted the data. NC drafted the manuscript and SMA put critical inputs in improving the draft. SMA was also involved in editing of the manuscript. Both authors read the final draft and approved it for submission.

## Pre-publication history

The pre-publication history for this paper can be accessed here:

http://www.biomedcentral.com/1471-2393/11/15/prepub
